# Re-analysis of the current status of clinical trial registration in China

**DOI:** 10.3389/fmed.2024.1394803

**Published:** 2025-01-31

**Authors:** Shuwen Li, Jianhao Li, Wenjing Zeng, Zhicheng Li, Jietong Zhang, Siying Wang, Nenggui Xu, Zeli Li

**Affiliations:** ^1^Dongguan Hospital of Guangzhou University of Chinese Medicine, Dongguan Traditional Chinese Medicine Hospital, Dongguan, China; ^2^Clinical Medical College of Acu-Moxi and Rehabilitation, South China Research Center for Acupuncture and Moxibustion, Guangzhou University of Chinese Medicine, Guangzhou, China; ^3^School of Computer, Electronics and Information of Guangxi University, Nanning, China; ^4^Guangzhou University of Chinese Medicine, Guangzhou, China

**Keywords:** current state of registration, Chinese clinical trial registry, evaluation of registration quality, re-analysis, insufficiency

## Abstract

**Objective:**

To reanalyze and summarize the current status and deficiencies of clinical trial registration in China, based on an analysis of the situation of clinical trial registration in the Chinese Clinical Trial Registry (ChiCTR).

**Methods:**

A search was conducted in China National Knowledge Infrastructure (CNKI), Chinese BioMedical Literature Database (CBM), Wangfang Data, VIP Database for Chinese Technical Periodicals (VIP), Web of Science (WoS), PubMed up to December 31, 2023, for literature on the analysis of clinical trial registration based on ChiCTR. NoteExpress software was used for screening, EXCEL for data organization and analysis, the Word Cloud website for constructing word frequency maps, and Origin software for visualization.

**Results:**

Among the 94 articles included, common analysis items included the number of registered projects, types of research, registration time, types of registration institutions, and regional distribution. Most of the included literature was funded. The publishing institutions involved 20 provinces/municipalities across the country, with hospitals being the majority of the publishing units. Most literature was submitted shortly after search completion and published shortly after submission, with more than half of the articles published in high-quality journals. The total average citation count of the literature was 3.34. The sources of clinical trial registration platforms analyzed in the literature were divided into three categories: single platform, dual platform, and multiple platforms.

**Conclusion:**

ChiCTR plays a key role in enhancing transparency in clinical research, promoting standardization and normalization of research, strengthening domestic and international scientific research cooperation, and advancing medical innovation and public health improvement. However, there are still issues with the quality of registration, focus areas, and the evaluation of registration quality.

## 1 Introduction

In recent years, the registration of clinical trials has become an important step in promoting transparency in clinical research, gradually gaining acceptance and importance among clinical researchers (1). As early as 2005, the World Health Organization (WHO) advocated that, based on scientific and ethical responsibilities and obligations, all clinical trials should be registered (2). The establishment of a clinical trial registration system ensures the transparency of trial designs and essential information, allowing the public to access information about clinical trials. This promotes the traceability of experimental results, reduces the likelihood of selective publication of results, and helps avoid duplicative research (1). Furthermore, as an obligation and responsibility of trial conductors, clinical trial registration can also facilitate the refinement and correction of experimental protocols during the registration and update process (3), thereby minimizing bias risk. Therefore, conducting clinical trial registration has become a necessary part of clinical research.

Following the 2004 declaration by the International Committee of Medical Journal Editors (ICMJE), which mandated that ICMJE member journals only publish results of clinical trials registered with a public clinical trial registry, China's West China Hospital established the Chinese Clinical Trial Registry (ChiCTR). In 2007, the Ministry of Health designated it as the national clinical trial registration center representing China. That same year, ChiCTR was accredited by the WHO as a primary registry in the international clinical trial registration platform. Currently, there is a substantial body of analyses based on randomized controlled trials (RCTs) registered with ChiCTR (1, 4–7). However, research from other countries has identified ongoing challenges in clinical trial registration, including non-standardized practices, inconsistent criteria for assessing registration quality, and insufficient focus on updating registration information, data sharing, and public transparency (8–10). In light of the limited attention these issues have received in China, this study re-analyzes the current state of ChiCTR-based research to assess the quality of clinical trial registrations. This aims to understand the impact of ChiCTR registration on standardizing clinical trials, address non-standard practices in ChiCTR registration, and propose recommendations for assessing the completeness and quality of clinical trial registrations.

## 2 Data and methods

### 2.1 Inclusion and exclusion criteria

#### 2.1.1 Inclusion criteria

① The research subject shall consist of an analysis of the registration status in the Chinese Clinical Trial Registry Center; ② The deadline for retrieval is December 31st, 2023; ③ Languages incorporated include Chinese and English literature.

#### 2.1.2 Exclusion criteria

① Exclude any literature that is not sourced from the Chinese Clinical Trial Registry; ② Exclude literature types that do not meet the requirements, such as Systematic reviews, Drug development, Manufacturing processes, Clinical trial protocols.

### 2.2 Literature search strategy

This study conducted a search through databases including China National Knowledge Infrastructure (CNKI), SinoMed (CBM), Wanfang Data, VIP Database, Web of Science (WoS), and PubMed, covering the period from the inception of each database to December 31, 2023. The search terms used were (“中国临床试验注册中心” OR “ChiCTR” OR “中国临床试验”) AND (“注册信息分析” OR “注册现状分析” OR “研究进展” OR “现状” OR “注册特征” OR “报告质量” OR “注册研究概况” OR “注册特点” OR “现况” OR “评价指标” OR “发展趋势” OR “注册概况” OR “数据分析” OR “项目分析”) in Chinese, and (“China Clinical Trial Registry” OR “Chinese Clinical Trial Registry” OR “ChiCTR”) AND (“registration information analysis” OR “registration status analysis” OR “research progress” OR “status” OR “registration characteristics” OR “report quality” OR “registration overview” OR “registration features” OR “current situation” OR “evaluation indicators” OR “development trends” OR “data analysis” OR “project analysis”) in English.

### 2.3 Data extraction

Using Excel software, Zhicheng Li and Jietong Zhang independently extracted the data from the included studies. In cases where there were discrepancies, they were resolved through discussion, with a third reviewer (Wenjing Zeng) consulted to reach a consensus if necessary. The Word Cloud 3.04 website (https://www.wenziyun.cn/ciyun/editor) is used to create a frequency visualization, along with Origin software for further visualization. Extracted information includes:

(1) Number of registered projects; (2) Type of research; (3) Registration time; (4) Type and geographical distribution of registering institutions; (5) Sample size; (6) Intervention measures; (7) Study design; (8) Sources of funding or materials; (9) Use of randomization and blinding; (10) Number of research centers; (11) Measurement indicators; (12) Ethical approval status; (13) Registration number status; (14) Phase of the study; (15) Recruitment of research subjects; (16) Classification of research diseases; (17) Implementation time of the study; (18) Sponsoring organization of the trial; (19) Primary measurement indicators; (20) Setup of Data and Safety Monitoring Board; (21) Informed consent signing status; (22) Age range of recruited research subjects; (23) Blinding method; (24) Measurement time points; (25) Completion of registration; (26) Side effect measurement indicators; (27) Disease staging; (28) Quality evaluation of registration content; (29) Quality evaluation of RCT; (30) Geographical distribution of study implementation; (31) Types of human specimens collected and their disposition; (32) Real-world study status; (33) Trial withdrawal status; (34) Intervention groups; (35) Statistical results of the study and the openness/sharing of original data; (36) Publication status of the registered project in Web of Science; (37) Secondary measurement indicators; (38) Measurement methods; (39) Distribution of Traditional Chinese Medicine (TCM) syndrome types; (40) Distribution of research fields; (41) “Indications” section in registration; (42) Level of implementing institutions; (43) Data collection and management methods; (44) Data disclosure timing.

## 3 Results

### 3.1 Results of literature search

Preliminary search results yielded 64 articles from CNKI, 135 from Wanfang Data, 613 from VIP, 186 from CBM, 795 from WoS, and 1,214 from PubMed. After using NoteExpress software to remove duplicates, 52 articles remained from CNKI, 78 from Wanfang Data, 375 from VIP, 49 from CBM, 679 from WoS, and 816 from Pubmed. Subsequent screening based on titles and abstracts for relevance to the topic reduced the numbers to 14 from CNKI, 82 from Wanfang Data, three from VIP, one from CBM, two from WoS, and three from Pubmed, totaling 105 articles. After a detailed selection process, 94 articles were ultimately included in the analysis.

### 3.2 Classification of study subjects in the included literature

**Figure 1:**
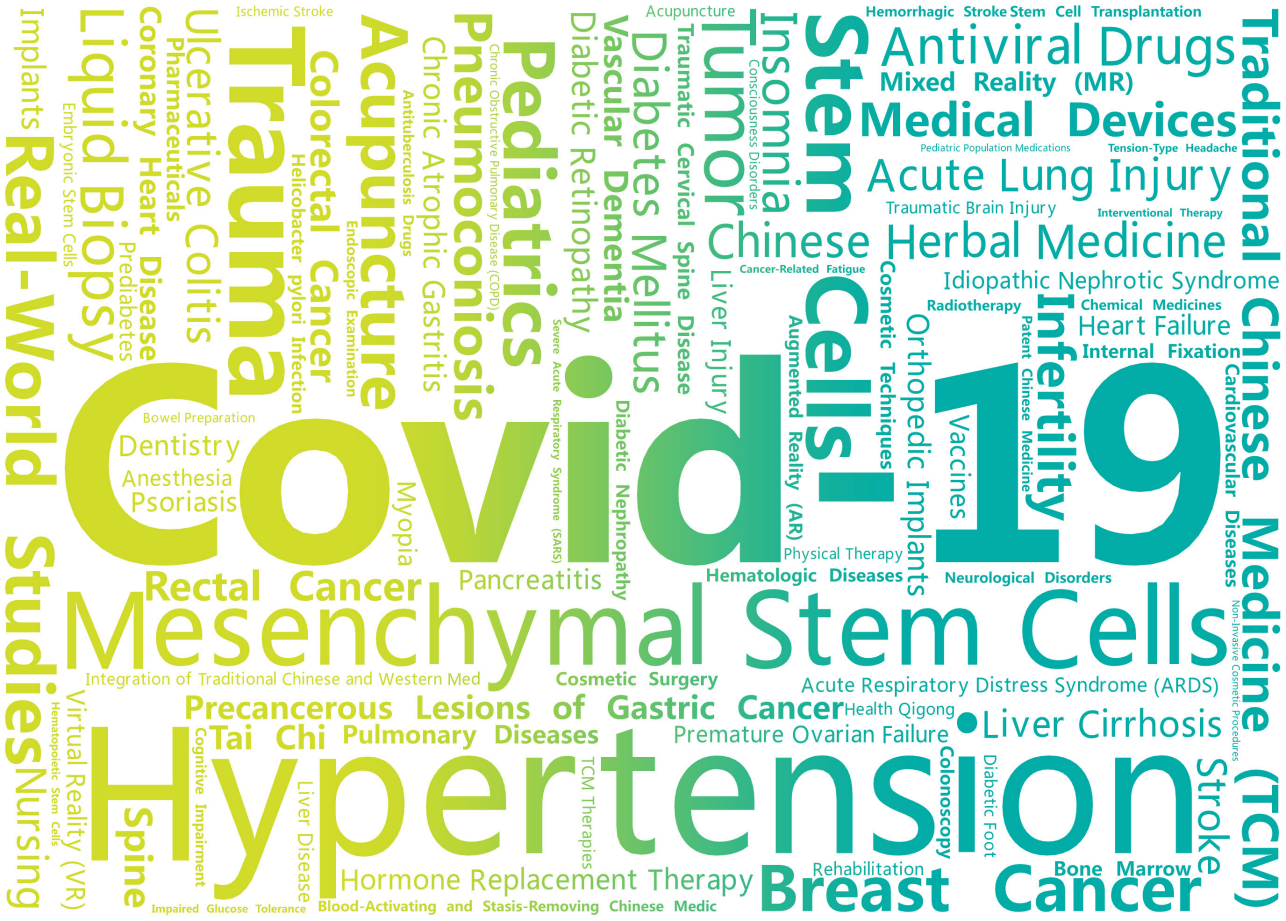
Keyword word cloud.

The study directions of articles analyzing the current status of clinical trial registration in China involve diseases, intervention measures, evaluation indicators, testing indicators, tumor screening technologies, real-world studies, etc.; the diseases studied include infectious diseases, digestive system diseases, cardiovascular diseases, etc., covering 14 major categories and a total of 41 diseases. The research directions include COVID-19 (27 times), diabetes and its complications (six times), hypertension (three times), real-world studies (three times), pneumoconiosis (two times), chronic atrophic gastritis (two times), breast cancer (two times), acupuncture (two times), and stem cells (two times). See Table 1 for details. Collect all keywords, select those related to diseases and intervention measures, and create a word cloud as shown in Figure 1.

**Table 1:** Classification of study subjects.

**Research subject categories**			**Frequency**	**Research subject categories**			**Frequency**
Disease categories	Infectious disease	COVID-19	27	Disease categories	Cancer-related diseases	Breast cancer	2
		H1N1	1			Colorectal cancer	1
		SARS	1			Rectal cancer	1
	Digestive system diseases	Liver injury	1			Cancer-related fatigue	1
		Liver cirrhosis	1		Obstetrics and gynecology	Premature ovarian failure	1
		Acute pancreatitis	1			Infertility	1
		Ulcerative colitis	1		Trauma-related conditions	Traumatic cervical spine injury	1
		Chronic atrophic gastritis	2			Traumatic brain injury	1
		Helicobacter pylori	1		Dentistry	Oral health conditions	1
	Cardiovascular system diseases	Hypertension	3		Ophthalmology	Myopia	1
		Coronary heart disease	1		Dermatology	Psoriasis	1
		Stroke	1		Urinary system conditions	Idiopathic nephrotic syndrome in children	1
		Hypertension in special populations	1	Intervention measures category	Acupuncture		2
		Heart failure	1		Stem cell therapy		2
		Cardiovascular diseases	1		Fitness Qigong		1
	Neurological diseases	Vascular dementia	1		Rehabilitative exercise therapy		1
		Consciousness disorders	1		Tai Chi		1
		Ischemic stroke	1		Digestive endoscopy		1
		Vascular cognitive impairment	1		Virtual, augmented, and mixed reality		1
		Tension headache	1		Cosmetic surgery medical devices		1
		Hemorrhagic stroke	1		Nursing care		1
	Endocrine system diseases	Diabetes	2		Radiation therapy		1
		Prediabetes	1		Anesthesia protocols		1
		Diabetic nephropathy	1	Others	Real-world studies		3
		Diabetic retinopathy	1		Chinese registry studies		1
		Diabetic foot	1		Traditional Chinese medicine research		1
	Respiratory system diseases	Pneumoconiosis	2		Evaluation indicators (COVID-19)		1
		Pulmonary diseases	1		Clinical trials (children)		1
		Acute lung injury/acute respiratory distress syndrome	1		Diagnostic indicators (diabetic nephropathy)		1
		COPD	1		Tumor screening techniques (liquid biopsy)		1
	Mental disorders	Insomnia	1		Bowel preparation (colonoscopy)		1

### 3.3 Analysis of entries in the included literature

The literature included in this study analyzed a total of 44 entries related to clinical trial registration, covering most of the registration items provided by the “China Clinical Trials Registry.” The number of entries analyzed per article ranged from a minimum of 2 to a maximum of 17, with an average of (9.37 ± 3.19) entries. The research frequency of the 44 study entries ranged from a minimum of once to a maximum of 89 times; the top 10 items by research frequency were the number of registered items, study types, registration time, types and regional distribution of registration institutions, sample size, intervention measures, study design, sources of funding or materials, use of randomization and blinding, and the number of research centers. Entries analyzed less frequently mainly focused on “ethical related,” “registration content quality evaluation,” and “data management related content.” See Table 2 for details.

**Table 2:** Research projects.

**Category**	**Research entries**	**Frequency**	**Category**	**Research entries**	**Frequency**
Registration status	Number of registered projects	89	Experimental design	Type of study	75
	Registration time	70		Sample size	61
	Registration status	27		Intervention measures	60
Observation indicators	Measurement indicators	31		Study design	57
	Primary measurement indicators	10		Use of randomization and blinding	41
	Measurement time points	5		Study implementation time	14
	Adverse effect measurement indicators	4		Recruitment age range of study subjects	7
	Types of human specimens collected and specimen disposition	3		Blinding method	6
	Measurement methods	1		Intervention group details	2
	Secondary measurement indicators	1		Registration column “indications” details	1
Study subjects	Disease classification for research	18	Related to research implementing entities	Type and geographic distribution of registering institutions	65
	Staging of diseases	4		Funding or material sources	49
	Distribution of research fields	1		Number of study centers	39
	TCM syndrome types	1		Trial sponsoring entity	14
Research progress	Stage of the research	25		Geographic distribution of study implementation	4
	Recruitment status of study subjects	25		Level of study implementing entities	1
	Trial withdrawal status	2	Data management	Setting up of data and safety monitoring board	10
Ethics related	Ethics approval status	31		Public disclosure/sharing of study statistical results and raw data	2
	Informed consent signing status	7		Data collection and management methods	1
Quality assessment	Registration completeness	5		Data disclosure timing	1
	Quality assessment of registration content	4	Others	Real-world study conditions	2
	Quality assessment of RCT	4		Publication status of registered projects in web of science	1

### 3.4 Funding support for the included literature

**Figure 2:**
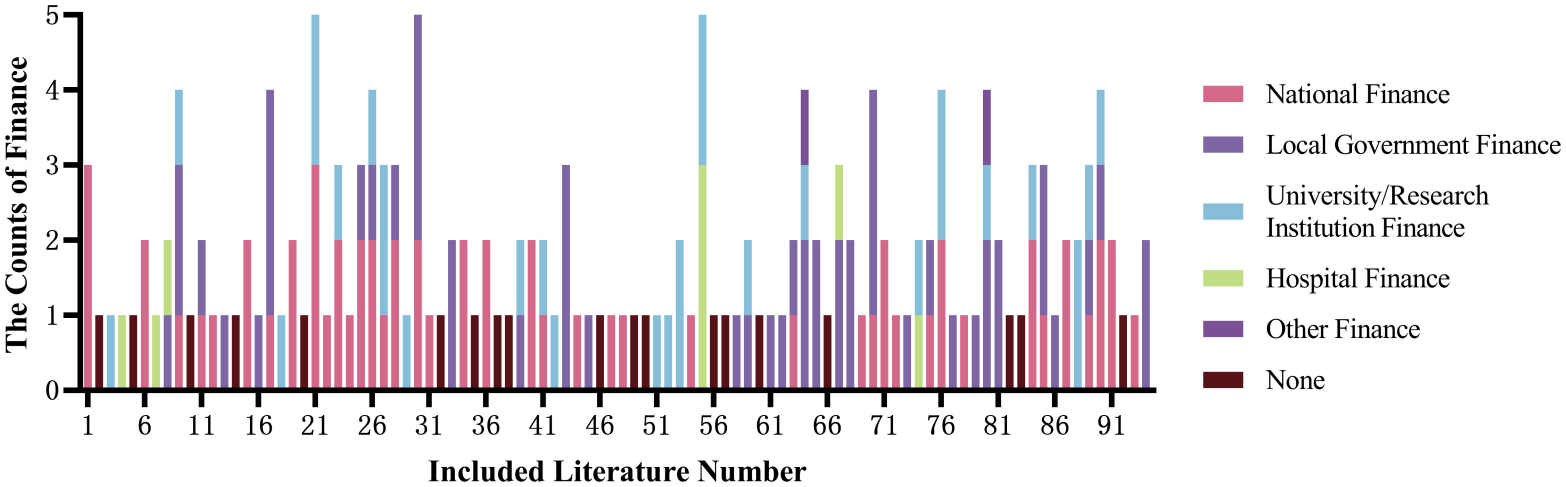
Finance sources for the included literature.

Out of the 94 studies included in the analysis, 75 items (79.79%) received funding support, while 19 items (20.21%) did not receive any funding; 45 items (47.87%) received multiple funding supports, among which 24 items (25.53%) received two types of funding, 10 items (10.64%) received three types, and 11 items (11.70%) received more than three types of funding. Among all funding sources, the top three were national finance, local finance, and universities/research institutes. See Table 3 and Figure 2 for details.

**Table 3:** Sources of research funding.

**Funding sources**	**Total quantity**	**Composition ratio (%)**
National finance	62	35.63
Local finance	53	30.46
University/research institute support	30	17.24
Hospital support	8	4.60
Others	2	1.15
None	19	10.92

### 3.5 Research institutions and regional distribution of literature

The literature included in this study involves publishing institutions from 20 provinces/municipalities and 25 cities/municipalities across the country, among which municipalities and provincial capitals account for 19 (76.00%). The top five cities in terms of publication volume are Beijing (27 times, 28.72%), Tianjin (11 times, 11.70%), Guangzhou (10 times, 10.64%), Shanghai (seven times, 7.45%), and Chengdu (seven times, 7.45%). There are 61 articles (64.89%) published by hospitals, 30 articles (31.91%) published by universities, and two articles and one article published by research institutes and disease control centers, respectively.

### 3.6 Comparison of publication timing in the included literature

The search results for submission intervals show that, shortly after the search was completed (within 3 months), there were 53 articles (56.38%) submitted, a longer time (3–6 months) saw 17 articles (18.09%) submitted, and a very long time later (more than 6 months) there were 14 articles (9.57%) submitted. There were three studies whose submission dates were earlier than the search dates (7–9). The interval from submission to being published online shows that, within a short time after submission (within 6 months), 60 articles (63.83%) were published online, and it took over a year for seven articles (7.45%) to be published online. See Table 4 for details.

**Table 4:** Comparison of search, acceptance, and online publication times.

**Time from retrieval to submission (months)**	**Total quantity**	**Composition ratio (%)**	**Time from submission to online publication (months)**	**Total quantity**	**Composition ratio (%)**
[0, 1]	35	37.23	[0, 3]	35	37.23
[1, 3]	18	19.15	(3, 6]	25	26.60
[3, 6]	17	18.09	(6, 9]	16	17.02
[6, 12]	8	8.51	(9, 12]	9	9.57
>12	6	6.38	>12	7	7.45
Submission predates retrieval cutoff	3	3.19	Unable to ascertain exact	2	2.13
Unable to ascertain exact time	7	7.45			

### 3.7 Journal analysis of the included literature

The 94 studies included in the analysis were published in 67 journals, both domestic and international, including five English-language journals and 62 Chinese-language journals. Among these, there are four SCI journals, four journals of excellence, 17 CSCD core journals, 21 core journals indexed by Peking University, six CSCD expanded journals, 50 science and technology core journals, and 12 non-core journals. High-quality journals (SCI, journals of excellence, CSCD, Peking University core, etc.) accounted for 54 publications (57.45%), core and above journals accounted for 80 publications (85.11%), and non-core journals accounted for 14 publications (14.89%). See Table 5 for details.

**Table 5:** Journal information and publication volume.

**Journal name**	**Level**	**Count**	**Journal name**	**Level**	**Count**
*BMC Med*	a(Q1)	1	*Journal of Integrated Traditional Chinese and Western Medicine Liver Diseases*	f	2
*Drug Des Devel Ther*	a(Q3)	1	*Journal of Traditional Chinese Medicine*	f	2
*J TRADIT CHIN MED*	a(Q3)	1	*China Medical Herald*	f	2
*Trials*	a(Q4)	1	*Hunan Journal of Traditional Chinese Medicine*	f	1
*Oncotarget*	/	1	*West China Medical Journal*	f	1
*Traditional Chinese Medicine Journal*	b, c, d, f	5	*Journal of Clinical Neurosurgery*	f	1
*China Journal of Traditional Chinese Medicine and Pharmacy*	b, c, d, f	2	*World Clinical Drugs*	f	1
*Biotechnology Bulletin*	b, c, d, f	1	*Tianjin Traditional Chinese Medicine*	f	1
*China Journal of Chinese Materia Medica*	b, c, d, f	1	*Journal of Yunnan University of Traditional Chinese Medicine*	f	1
*Chinese Journal of Evidence-Based Medicine*	c, d, f	6	*Chinese Journal of Leprosy and Skin Diseases*	f	1
*PLA Medical Journal*	c, d, f	2	*Chinese Journal of Evidence-Based Cardiovascular Medicine*	f	1
*Journal of Shanghai Jiao Tong University (Medical Science)*	c, d, f	2	*International Journal of Epidemiology and Infectious Diseases*	f	1
*Acta Pharmaceutica Sinica*	c, d, f	1	*International Journal of Traditional Chinese Medicine*	f	1
*Chinese Journal of Infection Control*	c, d, f	1	*Chinese Journal of Pediatrics of Traditional Chinese and Western Medicine*	f	1
*Chinese Journal of Modern Applied Pharmacy*	c, d, f	1	*Chinese Journal of Traditional Chinese Ophthalmology*	f	1
*Chinese Journal of Integrated Traditional and Western Medicine*	c, d, f	1	*Chinese Journal of Oncology Surgery*	f	1
*Chinese Oncology*	c, d, f	1	*Chinese Journal of Modern Nursing*	f	1
*Chinese Journal of Stomatology*	c, d, f	1	*Chinese Journal of Pancreatic Diseases*	f	1
*Chinese Journal of Clinical Pharmacology and Therapeutics*	c, f	2	*Journal of Heart, Brain and Blood Vessel Diseases of Integrated Traditional Chinese and Western Medicine*	f	1
*Chinese Journal of Infectious Diseases*	c, f	1	*Traditional Chinese Medicine Herald*	f	1
*Chinese Journal of Digestive Endoscopy*	c, f	1	*Chongqing Medicine*	f	1
*Chinese Journal of Plastic Surgery*	c, f	1	*World's Latest Medical Information Digest*	g	2
*Chinese Journal of Industrial Hygiene and Occupational Diseases*	d, e, f	1	*China Modern Doctor*	g	2
*World Science and Technology - Modernization of Traditional Chinese Medicine*	d, e, f	1	*Henan Journal of Preventive Medicine*	g	1
*Chinese Journal of Integrated Traditional and Western Medicine in Emergency*	d, e, f	1	*Pharmacy Today*	g	1
*Chinese Journal of Hospital Pharmacy*	d, f	2	*Southwest Medical University*	g	1
*Chinese Journal of Tissue Engineering Research*	d, f	2	*China Prescription Drug*	g	1
*Recent Advances in Ophthalmology*	d, f	1	*China Food and Drug Administration*	g	1
*China Pharmacy*	d, f	1	*Chinese Drug Evaluation*	g	1
*Chinese Hospital Management*	d, f	1	*Chinese Journal of Brain Diseases and Rehabilitation (Electronic Version)*	g	1
*Journal of Rehabilitation*	e, f	1	*Chinese Science and Technology Data (Full Text) Medicine and Health*	g	1
*Chinese Journal of Information on Traditional Chinese Medicine*	e, f	7	*Traditional Chinese Medicine and Clinical Pharmacology*	g	1
*Shanghai Journal of Traditional Chinese Medicine*	e, f	1	*Clinical Research in Traditional Chinese Medicine*	g	1
*World Traditional Chinese Medicine*	f	2			

### 3.8 Publication year and citation count of the included literature

**Figure 3:**
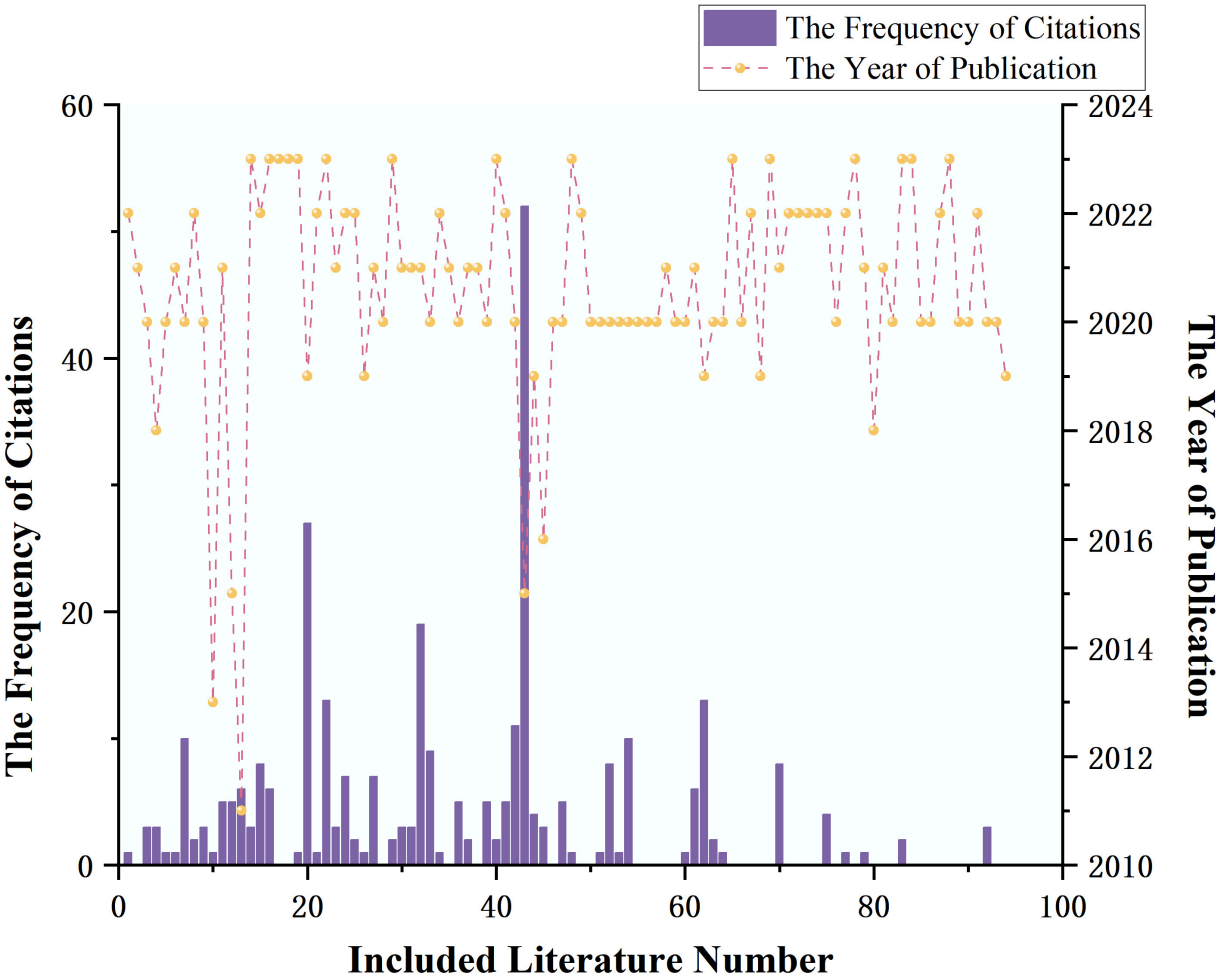
The frequency of citations and the year of publication in the literature.

Among the literature included in the analysis, the earliest was published in 2011, with a total of seven articles published in 2018 and before. The year with the highest number of publications was 2020, with 32 articles. The highest number of citations for a single article was 53, with 56 articles (59.57%) being cited at least once. The overall average citation count was 3.34 times, with the annual average citation counts for the years 2020, 2021, 2022, and 2023 being 6.41, 2.06, 1.60, and 0.33 times, respectively. See Figure 3 for details.

### 3.9 Sources and comparison methods of clinical trial registration platforms in the included literature analysis

The sources of clinical trial registration platforms analyzed in the literature included in this study are divided into three categories: single clinical trial registration platform, dual clinical trial registration platforms, and multiple clinical trial registration platforms; among them, single platform (all ChiCTR) accounts for 42 articles (44.68%), dual platforms (both ChiCTR and ClinicalTrials.gov) for 40 articles (42.55%), and multiple platforms (including ChiCTR and ClinicalTrials.gov among others recognized by the International Committee of Medical Journal Editors) for 12 articles (12.77%).

The comparison methods classified in the included literature analysis are categorized into seven main types: longitudinal comparison of a particular study subject within a single platform (ChiCTR, 42 articles, 44.68%); longitudinal comparison of a particular study subject within dual platforms only (ChiCTR and ClinicalTrials.gov, 32 articles, 34.04%); both longitudinal and cross-sectional comparison of a subject within dual platforms (ChiCTR and ClinicalTrials.gov, eight articles, 8.51%), including four articles as part of a review and one article compared with related high-impact factor publications; longitudinal comparison within multiple platforms of a particular study subject (including ChiCTR and ClinicalTrials.gov among others recognized by the International Committee of Medical Journal Editors, seven articles, 7.45%); both longitudinal and cross-sectional comparison of a subject within multiple platforms (five articles, 5.32%).

## 4 Discussion

As evidence-based medicine progresses, the significance of clinical trial registration is increasingly acknowledged for its critical role in enhancing research transparency, increasing public trust, fulfilling ethical duties, facilitating data sharing, and preserving the completeness of research (11, 12). Clinical trial registration has also become a valuable source of information for patients and their families. It provides them with a more comprehensive understanding of existing treatment options and medical advancements, enabling them to make more informed health choices (13).

At present, there exist numerous registration and filing information systems for medical research in China. One example is the International Traditional Medicine Clinical Trial Registry (ITMCTR), which was established in 2023 and certified by the WHO as a primary registry in the same year. It is noteworthy that this is the first clinical trial registry platform in the world that concentrates on traditional medicine (14). The Drug Clinical Trial Registration and Information Disclosure Platform (http://www.chinadrugtrials.org.cn/index.html) was established in 2012. This platform registers and discloses all clinical trials that are conducted in China, including bioequivalence trials, pharmacokinetic (PK) trials, Phase I, II, III, and IV trials (15). The Medical Research Registration Information System (https://www.medicalresearch.org.cn) comprises two subsystems: one for stem cell clinical research institutions information and the other for medical research project registration. The medical research project registration subsystem is utilized to register clinical medical research projects (16). Moreover, there are several clinical registration platforms overseas, such as the National Institutes of Health (NIH) Clinical Trials Registry (http://www.clinicaltrials.gov), and the International Standard Randomized Controlled Trial Number Registry (http://isrctn.org), the Dutch Trial Register (www.trialregister.nl). The ChiCTR, which is the focus of this study, is particularly representative.

By centralizing the management of clinical trial information, ChiCTR not only ensures high-quality research but also accelerates the widespread dissemination. In recent years, there has been a significant increase in the number of research projects registered with ChiCTR. This study includes a total of 94 articles in both English and Chinese, covering various aspects such as disease types, intervention measures, and trial methods. Among these, COVID-19 was the most frequently mentioned condition. The interventions involved acupuncture and stem cell therapy, with real-world studies being the predominant trial methodology. A significant number of clinical trial registrations for COVID-19 were recorded, highlighting the extensive use of ChiCTR during the pandemic. This facilitated global sharing of scientific discoveries and research outcomes related to the coronavirus. An increasing number of studies have analyzed ChiCTR registration data, reflecting a growing awareness of clinical trial registration in China. However, certain shortcomings still persist.

This study found that ChiCTR primarily focuses on aspects such as study type, registration time, institution type and regional distribution, sample size, interventions, study design, funding sources, application of randomization and blinding, and the number of research centers. However, ChiCTR appears to overlook some critical areas, such as updating study progress, managing data, addressing ethical issues, and evaluating the quality of registered content, all of which are essential for ensuring the quality of clinical trials and upholding scientific integrity (17, 18). Therefore, to further enhance the quality and systematic standards of clinical trial registration in ChiCTR, it is essential to explore the current status of these key issues and propose targeted improvements.

For assessing the quality of clinical trial registration content, it is recommended to establish an evaluation framework based on international standards such as WHO registration criteria or ICMJE guidelines. This framework should include indicators for registration completeness, timeliness, consistency, disclosure, and compliance with standards, with regular evaluations conducted. A scoring system can be adopted to grade quality based on the number of completed items and the level of detail provided, categorizing registrations as “excellent,” “adequate,” or “incomplete,” and this classification should be clearly displayed on the registration page.

Additionally, periodic checks can be carried out to assess the frequency of updates and ensure consistency between registered information and published results. Reminders can be sent based on the registration completion date, encouraging registrants to keep their data up to date and disclose trial results promptly. Modern communication tools, such as WeChat, mini-programs, and email notifications, can facilitate these reminders and promote timely updates. Furthermore, data sharing can be incentivized through requirements set by journals or funding agencies. Regular publication of registration quality assessments can also increase transparency, motivating researchers and institutions to improve registration practices.

Properly designed trials can enhance research efficiency and reduce the likelihood of failure (19). However, due to the lack of standardized guidelines, the quality of trial designs varies widely, affecting the reliability and comparability of research outcomes. Therefore, establishing registration standards and guidelines is crucial. By referencing internationally recognized standards, clear guidelines can be developed to ensure that accurate information is provided during registration, such as the definition of primary and secondary outcomes, sample size calculation methods, and trial protocol details. Additionally, a quality review process for registration information should be introduced, combining manual review with automated tools to improve efficiency and ensure compliance with standards. Moreover, providing training on clinical trial registration for researchers can help them understand the importance of registration requirements and standardization, thereby improving the quality and consistency of submitted information. Regulatory authorities should also enhance oversight and guidance in these critical areas by establishing and refining relevant policies and procedures, such as requiring regular updates on study progress, implementing stringent data management standards, standardizing ethical review processes, and conducting quality assessments of registration content. These measures are essential for improving the overall quality of clinical trials in China and boosting the country's international reputation in the field.

The literature analyzed in this study largely received funding from national and local governments, as well as universities and research institutes, with the publishing institutions mainly located in first-tier cities and primarily consisting of hospitals and universities. This finding reveals the characteristics of funding support and institutional distribution in Chinese clinical trial research, highlighting several key trends: First, the main sources of funding are government and academic institutions, underscoring the vital role of public funds in driving medical research, ensuring the continuity of studies, and the production of outcomes. Second, the concentration of research institutions in resource-rich first-tier cities, while fostering an efficient research environment, also exposes the issue of uneven regional resource distribution. Lastly, the role of hospitals and universities as the main publishers emphasizes their central role in cultivating research talent and driving medical innovation.

To enhance research quality and impact, the following suggestions are crucial: diversify funding sources by attracting more non-governmental funding, such as corporate and private investment, to enrich the research funding pool and stimulate innovation; promote inter-regional cooperation by establishing collaborative platforms for resource sharing, thus narrowing the development gap between regions; and strengthen interdisciplinary cooperation by encouraging interactions between hospitals, universities, and other institutions, fostering integration of medical research with other disciplines, and exploring new research paths. Implementing these strategies can optimize resource allocation, and improve research efficiency and outcomes.

Furthermore, the analysis of literature also exposes a deficiency in the recognition of pharmaceutical companies' contributions as a major source of clinical research funding. These companies invest significant amounts of money, particularly in drug clinical trials, yet their efforts are often overlooked due to a lack of acknowledgment in the literature.

The analysis of this study found that the majority of the included literature was submitted within 3 months after the search was completed, and the cycle from submission to publication generally did not exceed 6 months. These studies are often published in high-impact journals and generally have a high frequency of citations. The clinical trial registration platforms involved include single, dual, and multiple platforms, and the comparison methods used cover longitudinal, cross-sectional, and a combination of both, reflecting the diversity and innovation of research design and analysis methods. This result highlights the comprehensive performance of Chinese clinical trial research in terms of publication efficiency, quality, and impact, while also showcasing the ability to timely share results with the global scientific community, promoting the update and dissemination of medical knowledge. The acceptance and publication of these studies in high-quality journals signify that they meet the high standards of the international scientific community for analysis depth and result interpretation, enhancing the international reputation of Chinese clinical trial research and increasing academic influence in the field. The diversity of research methods not only helps in deeply evaluating and interpreting clinical trial results, enhancing the scientific nature and accuracy of the research but also guides future research directions, providing new ideas and scientific evidence for researchers, advancing medical science.

However, it's important to emphasize that while rapid publication and publication in high-impact journals are commendable goals, the quality of the research, ethical standards, and social value remain the core of scientific work. Therefore, maintaining high-quality research standards and ensuring the ethicality and reliability of studies are crucial for the continued development of health sciences, even as we pursue speed and impact.

There are several limitations in this study. This study solely analyzed randomized controlled trial literature registered in ChiCTR, which may have overlooked pertinent studies registered on other clinical registration platforms in China and other nations, resulting in incomplete analysis. Moreover, this study solely searched six databases and was confined to Chinese or English languages, which may have led to the omission of relevant literature. We cannot assess the accuracy of the clinical studies included in the analysis, as it depends on the availability of publicly accessible sources of literature. Additionally, while ChiCTR continues to update and improve, providing transparency for the types, designs, distribution, and funding of clinical trials in China, it remains unclear whether the quality of this study can promote and improve the dissemination of clinical registration in China. While we propose developing a standard, creating a comprehensive assessment standard requires extensive research methodology, iterative expert consultations, and significant time, as it encompasses a scope far beyond this manuscript. We are actively pursuing this endeavor, but it remains an ongoing project.

In conclusion, the current state of clinical trial registration in China presents a number of issues. The most significant problem is the presence of multiple regulatory authorities and the absence of a unified and mandatory set of regulations to standardize and guide the process. Due to varying regulatory standards and requirements across different departments, the registration process of clinical trials is fraught with uncertainty. Researchers must devote significant time and effort to understanding the regulations and requirements of each department to ensure the smooth conduct of clinical trials. This inevitably increases the burden on researchers and impacts the progress and quality of clinical trials.

Furthermore, Good Clinical Practice (GCP) is described as a collection of guidelines for the planning, execution, monitoring, termination, inspection, reporting, and documentation of clinical trials. The primary goal is to ensure adherence to scientific and ethical principles and the proper documentation of the investigational product's (diagnostic, therapeutic, or preventive) characteristics. The aim is to guarantee the dependability and validity of the clinical trial process while safeguarding the rights and safety of the trial participants. During the process of registering clinical trials, there has been an increasing emphasis on GCP in China, which highlights the country's commitment to enhancing the quality of clinical trials. However, the literature analyzed in this study did not provide a comprehensive description or analysis of GCP items. Therefore, we recommend that future articles related to clinical trials should include detailed descriptions of GCP items to ensure the quality and integrity of the research.

In summary, the lack of unified and mandatory regulations in China has resulted in a casual and chaotic approach to the registration of clinical trials. Some researchers may choose to register only on certain platforms for various reasons, neglecting other important registration platforms. This not only leads to the dispersion and fragmentation of information but also makes it difficult for regulatory authorities to fully understand the overall situation of clinical trials in China. In the past, many Chinese authors only registered on ClinicalTrials.gov, which has resulted in certain issues. Firstly, it has led to incomplete and inaccurate collection and organization of clinical trial information in China. Secondly, it has resulted in the neglect or omission of some important clinical trials, thereby affecting the overall level and quality of clinical trials in China.

## 5 Conclusion

This study examines the current status of RCT studies based on ChiCTR and specifically analyzes the research community's understanding of clinical trial registration and their perception of the advantages and disadvantages of the ChiCTR system. The study highlights significant achievements in funding acquisition, the geographical distribution of research institutions, and the speed and quality of research output. However, it also notes that researchers tend to focus more on experimental design rather than giving sufficient attention to key aspects such as real-time updates on research progress, effective data management, in-depth consideration of ethical issues, and systematic evaluation of registration information. Additionally, unequal distribution of resources among regions has become an important factor constraining overall scientific progress.

There are certain limitations and constraints that must be acknowledged in this study. Firstly, the study solely relied on literature from RCTs registered on the ChiCTR platform for further analysis, potentially excluding relevant literature from other clinical registration platforms in China and abroad, thereby resulting in an incomplete analysis. Secondly, we were unable to evaluate the accuracy of the clinical studies included in the analysis due to the unavailability of publicly accessible sources of literature. Lastly, while ChiCTR continues to update and improve, it remains unclear whether this study's quality can contribute to promoting and enhancing the dissemination of clinical registration in China by providing transparency on the types, designs, distribution, and funding of clinical trials.

## Data Availability

The original contributions presented in the study are included in the article/Supplementary material, further inquiries can be directed to the corresponding author.
