# Supplementary material for: Re-analysis of the current status of clinical trial registration in China
Source: Front Med (Lausanne). 2025 Jan 31;11:1394803. doi: 10.3389/fmed.2024.1394803 (PMC11826807; doi:10.3389/fmed.2024.1394803)
Supplement: Supplementary file 1 [file Table_1.DOCX]

Supplementary

List of citation for all articles ultimately included in the analysis

| No | Article Title in Quotation Marks |
| --- | --- |
| 1 | Mao J, Lin S, Wang J, et al. A review of the registration of clinical trials on traditional Chinese medicine for cardiovascular diseases from 2007 to 2020. Chin J Tradit Chin Med. 2022, 63(07): 601-607. |
| 2 | Zhu B, Liu Y, Li J, et al. Quality assessment of psoriasis clinical trials in China from 2016 to 2020. Chin J Lepr Dermatol. 2021, 37(12): 779-782. |
| 3 | Hou F, Liu J, Bai Z, et al. Analysis of the registration status of clinical studies on COVID-19. J PLA Med J. 2020, 45(04): 365-369. |
| 4 | Yang L, Chen S, Yang D, et al. A quality analysis of clinical anaesthesia study protocols from the Chinese clinical trials registry according to the SPIRIT statement. Oncotarget. 2018, 15, 9(37): 24830-24836. |
| 5 | Lu L, Li F, Wen H, et al. An evidence mapping and analysis of registered COVID-19 clinical trials in China. BMC Med. 2020, 18(1): 167. |
| 6 | Kuang ZR, Li XY, Cai JX, et al. Calling for improved quality in the registration of traditional Chinese medicine during the public health emergency: a survey of trial registries for COVID-19, H1N1, and SARS. Trials. 2021, 22(1): 259. |
| 7 | Huang JH, He YC, Su QM, et al. Characteristics of COVID-19 Clinical Trials in China Based on the Registration Data on ChiCTR and ClinicalTrials.gov. Drug Des Devel Ther. 2020, 14: 2159-2164. |
| 8 | Wei JJ, Guo RJ, Fu GJ, et al. Registration of intervention trials of Traditional Chinese Medicine for four neurological diseases on Chinese Clinical Trial Registry and ClinicalTrials.gov: a narrative review. J Tradit Chin Med. 2022, 42(1): 148-153. |
| 9 | Zhao H, Xie Y, Li J. Analysis of registration data of clinical trials on pneumoconiosis. Chin J Ind Hyg Occup Dis. 2020, 38(01): 20-23. |
| 10 | Nan J, Yuan H, Li K, et al. Analysis of registered clinical trial projects related to internal fixation implantation for traumatic cervical spine injuries. Chin J Tissue Eng Res. 2013, 17(22): 4115-4122. |
| 11 | Xue J, Jiang F, Liu H, et al. Analysis of the current status of registered clinical trials on liver injury. J Integr Tradit Chin West Med Liver Dis. 2021, 31(07): 629-634. |
| 12 | Liu H. Stem cells and stem cell therapy for diabetes: analysis of clinical trial registration information in China. Chin J Tissue Eng Res. 2015, 19(06): 975-979. |
| 13 | Guo Y, Shen X, Wang W, et al. Research progress on the use of stem cells for the treatment of breast cancer. Biotechnol Bull. 2011, (12): 43-50. |
| 14 | Zhang C, Huang J, Xu L, et al. Analysis of registration status of traditional Chinese medicine-related clinical trials for cancer-related fatigue in China. J Yunnan Univ Tradit Chin Med. 2023, 46(02): 106-110. |
| 15 | Ni W, Cao K, Ren B, et al. Analysis of registration status of clinical trials on vascular dementia in China. Chin J Tradit Chin Med Inf. 2022, 29(10): 53-58. |
| 16 | Tian X, Sun X, Ma T, et al. Analysis of registration status of traditional Chinese medicine clinical trials for hypertension prevention and treatment in China. Chin J Tradit Chin Med Inf. 2023, 30(03): 25-30. |
| 17 | Liang B, Lu Y, Yin X, et al. Analysis of registration status of traditional Chinese medicine clinical trials for ulcerative colitis in China. Chin J Tradit Chin Med Inf. 2023, 30(10): 63-68. |
| 18 | Sun X, Miao D, Tian X, et al. Analysis of the registration status of traditional Chinese medicine clinical trials for diabetes in China. Chin J Tradit Chin Med Inf. 2023, 30(01): 61-66. |
| 19 | Chen J, Ma X, Xu H, et al. Analysis of the registration status of clinical trials on blood-activating and stasis-eliminating Chinese medicines. J Integr Tradit Chin West Med Cardiocerebrovasc Dis. 2023, 21(05): 769-774. |
| 20 | Zhang T, Li W, Chen L. Analysis of the registration status of hypertension clinical trials in China based on ClinicalTrials.gov and the Chinese Clinical Trial Registry. West China Med J. 2019, 34(04): 419-424. |
| 21 | Liang Q, Yang Y, He J, et al. Analysis of the status of clinical trials on consciousness disorders over the past five years based on ClinicalTrials and ChiCTR. J Clin Neurosurg. 2022, 19(04): 402-410+416. |
| 22 | Chen L, Situ B, Mei Z, et al. Analysis of the status of infertility clinical trials in China based on domestic and international clinical trial registration platforms. Chin J Hosp Pharm. 2023, 43(20). |
| 23 | Li J, Zhao H, Xie Y. Considerations on research related to the prevention and treatment of pneumoconiosis based on clinical trial registration. Chin J Integr Tradit West Med. 2021, 41(10): 1255-1259. |
| 24 | Wang H, Feng Y, Yu Y, et al. Characteristics of rehabilitation exercise therapy for post-stroke recovery: an analysis over the past 20 years based on clinical trial registration platforms. J Rehabil. 2022, 32(05): 455-461. |
| 25 | Liu J, Huang X, Zhan H, et al. Analysis of the registration status of traditional Chinese medicine clinical trials based on registration platforms. Chin J Food Drug Regul. 2022, (07): 50-57. |
| 26 | Wang H, Hu H, Ji Z, et al. Analysis of the current status of real-world studies on traditional Chinese medicine based on clinical trial registration information. World Chin Med. 2019, 14(12): 3127-3134. |
| 27 | Xiong J, Tian Z, Wang F, et al. Analysis of the characteristics of registered clinical trials on chronic obstructive pulmonary disease based on bibliometric data. Hunan J Tradit Chin Med. 2021, 37(01): 120-122+134. |
| 28 | Liu R, Guo M, Zhang H, et al. Analysis of registered clinical studies on integrated traditional Chinese and Western medicine for COVID-19. World Latest Med Inf. 2020, 20(A4): 308-313. |
| 29 | Wang S, Li H, Ma W, et al. Analysis of the current status of real-world studies based on the Chinese Clinical Trial Registry. Chin J Evid-Based Med. 2023, 23(01): 75-79. |
| 30 | Zhou J, Li L, Deng W, et al. Evaluation of the characteristics and report quality of registered clinical trials on health Qigong. Chin J Evid-Based Med. 2021, 21(02): 197-203. |
| 31 | Miao Q, Wang B, Zhang H. Overview of registered clinical trials on traditional Chinese medicine for lung diseases over the past decade. J Tradit Chin Med. 2021, 62(22): 1933-1939. |
| 32 | Tan S. A randomized controlled study on the impact of cartoon educational brochures on patient bowel preparation [D]. Southwest Medical University, 2021. |
| 33 | Liu C, Jin X, Wang H, et al. Analysis of the registration status of clinical study protocols for antiviral drugs in the treatment of COVID-19. Chin J Integr Tradit West Med Emerg. 2020, 27(2): 176-185. |
| 34 | Tang J, Li J. Analysis of the characteristics and report quality of registered clinical trials on chronic atrophic gastritis. Clin Res Tradit Chin Med. 2022, 14(19): 131-135. |
| 35 | Du S, Guo S, Fang S, et al. Analysis of the registration status of traditional Chinese medicine clinical trials for chronic atrophic gastritis. Chin J Tradit Chin Med Inf. 2021, 28(07): 43-46. |
| 36 | Zhang C, Chen S, Zhang J, et al. An overview of registered clinical trial drugs for COVID-19 treatment. Acta Pharm Sin. 2020, 55(03): 355-365. |
| 37 | Wang X, Zheng P, Sun S. Analysis of the registration status of clinical trials on idiopathic nephrotic syndrome in children worldwide. Chin J Integr Tradit West Med Pediatr. 2021, 13(06): 480-484. |
| 38 | Shen X, Yang Y, Zhang R, et al. Characteristics of global clinical trial registrations for COVID-19. Chin J Infect Dis. 2021, 39(8): 464-469. |
| 39 | Qu T, Feng T, Jiang J, et al. Analysis and considerations on the registration information of global clinical studies for COVID-19. J Shanghai Jiao Tong Univ (Med Sci). 2020, 40(06): 707-712. |
| 40 | Wang Z, Tang T, Shen C, et al. Evaluation of the characteristics and reporting quality of registered clinical trials related to Tai Chi. Int J Tradit Chin Med. 2023, 45(8): 1027-1033. |
| 41 | Ju D, Wang X, Yu M, et al. Analysis of measurement indicators for diabetic nephropathy clinical trials registered in the Chinese Clinical Trial Registry. Med Health Care. 2022, (5): 118-121. |
| 42 | Zhang X, Zhao C, Sun Y, et al. Promoting the establishment of a collaboration and sharing mechanism for clinical trials—strategic thinking during the pandemic. J Tradit Chin Med. 2020, 61(08): 650-654. |
| 43 | Feng X, Sun X, An X, et al. Analysis of the registration status of pediatric clinical trials in China. Chin J Drug Eval. 2015, 32(04): 233-236. |
| 44 | He Y, Fan Z, Li J, et al. Current status of breast cancer clinical trial registration and publication of related papers in China. Chin Hosp Manag. 2019, 39(06): 57-59. |
| 45 | Yan H, Guan L, Cai H, et al. Analysis of the current status of digestive endoscopy registration studies in China. Chin J Dig Endosc. 2016, 33(11): 774-777. |
| 46 | Yin H, Zhang T, Li C, et al. Characteristics of clinical trials for COVID-19 in China. Chin J Infect Control. 2020, 19(08): 671-678. |
| 47 | Mu X, Zhao Y, Zhang L, et al. An overview of the current status of clinical drug research for the treatment of COVID-19 in China. Chin J Clin Pharmacol Ther. 2020, 25(08): 869-877. |
| 48 | Tan X, Wang M, Wang X, et al. Analysis of the registration status of clinical trials on traditional Chinese medicine treatments for insomnia in China. J Tradit Chin Med Guide. 2023, 29(4): 171-175. |
| 49 | Yang J, Zhang Y, Gao S, et al. Analysis of the registration status of traditional Chinese medicine clinical trials for coronary heart disease in China. Chin J Tradit Chin Med Inf. 2022, 29(03): 47-52. |
| 50 | Song G, Cheng M. Analysis of the characteristics of COVID-19 clinical trial registration: based on ClinicalTrials.gov and the Chinese Clinical Trial Registry. Chin Med Guide. 2020, 22(06): 369-374. |
| 51 | He Q, Kuang J, Tao M, et al. Analysis and considerations on the registration status of clinical research for COVID-19 in China. Tradit Chin Med Clin. 2020, 11(05): 1-4. |
| 52 | Yao C, Zhao W. Insights and the concept of a national clinical trial network from COVID-19 clinical research. Chin J Evid-Based Med. 2020, 20(05): 497-503. |
| 53 | Xu M. Analysis of the characteristics of COVID-19-related clinical trial registration information. Henan J Prev Med. 2020, 31(10): 728-731+762. |
| 54 | Zhai Y, Huang Q, Wu M, et al. Analysis and considerations on the registration projects for the prevention and treatment of COVID-19. Chin J Mod Appl Pharm. 2020, 37(4): 385-389. |
| 55 | Jin X, Pang B, Wang H, et al. Evaluation indicators and related issues of clinical trials for COVID-19. Tianjin J Tradit Chin Med. 2020, 37(10): 1109-1113. |
| 56 | Hou F, Liu J, Bai Z, et al. Analysis of the registration status of clinical studies on COVID-19. J PLA Med J. 2020, 45(04): 365-369. |
| 57 | Chen Y, Yang Z, Chen J, et al. Status and research progress of drug clinical trials for COVID-19. Pharm Today. 2020, 30(03): 160-163. |
| 58 | Yu F, Xin M, Liu N, et al. Problems and countermeasures in traditional Chinese medicine clinical research for COVID-19. J Tradit Chin Med. 2021, 36(04): 683-686. |
| 59 | Li S, Wang Y, He L, et al. Analysis of registered clinical trials for COVID-19 and existing problems and suggestions. J Tradit Chin Med. 2020, 61(23): 2025-2030. |
| 60 | Zhao S, Zhang B, Chang X, et al. Analysis of registered drug clinical studies for COVID-19 pneumonia. Chin J Hosp Pharm. 2020, 40(14): 1499-1504. |
| 61 | Dong W, Wen T, Wang W, et al. Analysis and considerations on the registration projects of COVID-19 vaccine clinical trials. Chin J Prescr Drugs. 2021, 19(11): 1-5. |
| 62 | Jia S, Xie L, Li L, et al. Current status of research on the application of liquid biopsy technology for cancer screening. Chin J Oncol. 2019, 28(10): 774-779. |
| 63 | Zhang Q, Meng X. Analysis of registered clinical trials for COVID-19 during the pandemic. World Clin Drugs. 2020, 41(05): 399-404. |
| 64 | Liu H, Jiang F, Xue J, et al. Analysis of the registration status of clinical trials for Helicobacter pylori infection. J Tradit Chin Med. 2020, 61(22): 1962-1966. |
| 65 | Gao J, Sun Y, Shu W, et al. Analysis of the current status of anti-tuberculosis drug clinical trials led by China. Int J Epidemiol Infect Dis. 2023, 50(02): 92-97. |
| 66 | Sun M, Xie Y. Analysis of clinical studies on approved traditional Chinese medicine for COVID-19 treatment based on registered trials. World Sci Technol Mod Tradit Chin Med. 2020, 22(03): 612-621. |
| 67 | Zeng C, Fang Y, Fan X. Investigation and analysis of the characteristics of MSC clinical trial registration in China. Chin J Mod Doctor. 2022, 60(29): 6-10. |
| 68 | Zhou L, Ouyang W, Li G, et al. Analysis of the current status of registered studies in China. Chin J Evid-Based Med. 2019, 19(06): 702-707. |
| 69 | Zhao J, Dai J, Yan Y, et al. Analysis of the registration status of clinical trials on liver cirrhosis in China. J Integr Tradit Chin West Med Liver Dis. 2023, 33(02): 135-137+141. |
| 70 | Jiang J, Jiang X, Zeng X, et al. Analysis of the current status of clinical research registration for acute pancreatitis in China. Chin J Pancreatol. 2021, 21(04): 251-257. |
| 71 | Lin Y, Zhang Y, Xia J, et al. Characteristics of registered clinical trials on traumatic brain injury in the Chinese Clinical Trial Registry. Chin J Brain Dis Rehabil (Electronic Edition). 2022, 12(2): 74-80. |
| 72 | Chen Y, Li Y, Guo N, et al. Analysis of the characteristics of registered projects on acute lung injury/acute respiratory distress syndrome in the Chinese Clinical Trial Registry. Chin Med Guide. 2022, 19(31): 154-158. |
| 73 | Zhang X, Chen X, Xia J, et al. Analysis of the characteristics of oral clinical research projects in the Chinese Clinical Trial Registry. Chin J Stomatol. 2022, 57(09): 946-952. |
| 74 | Chen Y, Li Y, Li X, et al. Analysis of the characteristics of virtual reality, augmented reality, and mixed reality projects in the Chinese Clinical Trial Registry. Chongqing Med. 2022, 51(04): 697-701. |
| 75 | Shi X, Zhang D, Luo X, et al. Analysis of the characteristics of pre-diabetes clinical trials in the Chinese Clinical Trial Registry. Chin J Mod Doctor. 2022, 60(06): 123-127. |
| 76 | Liu C, Li B, Zhang L, et al. Analysis of the characteristics of acupuncture clinical trials in the Chinese Clinical Trial Registry. World Latest Med Inf (Continuing Electronic Periodical). 2020, 20(45): 11-13. |
| 77 | Zeng M, Hong Z, Zhou R, et al. Analysis of the current status of registered myopia clinical trials in the Chinese Clinical Trial Registry. Adv Ophthalmol. 2022, 42(11): 900-905. |
| 78 | Wang J, Ma K, Liu Y, et al. Analysis of the characteristics of registered clinical trials on premature ovarian insufficiency in the Chinese Clinical Trial Registry. J Tradit Chin Med. 2023, 38(05): 1119-1124. |
| 79 | Wu H, Zhou S, Zhang Q, et al. Analysis of the characteristics of rectal cancer clinical trials in the Chinese Clinical Trial Registry. Chin J Oncol Surg. 2021, 13(04): 356-361. |
| 80 | Su Y, Quan L, Yang Z, et al. Analysis of the characteristics of hypertension clinical trials registered in the Chinese Clinical Trial Registry. Chin J Evid-Based Med. 2018, 18(10): 1114-1117. |
| 81 | Zhao Y, Li M, Ji H, et al. Analysis of the characteristics of nursing-related clinical trials registered in the Chinese Clinical Trial Registry. Chin J Mod Nurs. 2021, 27(03): 323-327. |
| 82 | Zhang X, Zhang H, Zhao X, et al. Analysis of the characteristics of acupuncture clinical trials registered in the Chinese Clinical Trial Registry. Chin J Tradit Chin Med. 2020, 35(04): 2078-2080. |
| 83 | Jiao W, Liu K, Shen W, et al. Analysis of the characteristics of real-world studies on traditional Chinese medicine registered in the Chinese Clinical Trial Registry. World Chin Med. 2023, 18(01): 118-126. |
| 84 | Zeng M, Liao B, Luo X, et al. Analysis of the registration status of clinical trials for diabetic retinopathy in China. Chin J Tradit Chin Med Ophthalmol. 2023, 33(07): 695-700. |
| 85 | Zhong M, Su Y, Guo R, et al. Analysis of the registration status of hypertension clinical trials in special populations in China. Chin J Evid-Based Med. 2020, 20(03): 325-329. |
| 86 | Xiang Y, Zeng C, Huang Z, et al. Analysis of the registration information of COVID-19 clinical trials in China. Chin J Clin Pharmacol Ther. 2020, 25(02): 135-140. |
| 87 | Che Y, Zheng Y, Wang Y, et al. Characteristics and development trends of registered clinical studies on plastic surgery and cosmetic medical devices in China. Chin J Plast Surg. 2022, 38(12): 1390-1396. |
| 88 | Cui H, Sun X, Zhang N, et al. Analysis of the registration status of clinical trials on traditional Chinese medicine for the prevention and treatment of colorectal cancer. Chin J Tradit Chin Med Inf. 2023, 30(07): 51-55. |
| 89 | Deng H, Zhao Y, Xu J, et al. Report on the dynamics of registered evidence-based studies of traditional Chinese medicine for the prevention and treatment of COVID-19. Shanghai J Tradit Chin Med. 2020, 54(03): 14-15. |
| 90 | Wang H, Jin X, Pang B, et al. Analysis of clinical research protocols for traditional Chinese medicine interventions in COVID-19 treatment. Chin J Chin Mater Med. 2020, 45(06): 1232-1241. |
| 91 | Dai X, Yang Q, Zhang W, et al. Analysis of registered clinical research protocols on traditional Chinese medicine for diabetic foot treatment. Chin J Evid-Based Cardiovasc Med. 2022, 14(08): 905-909. |
| 92 | Yao H, Zhao T, Ren X, et al. Overview of the registration status of clinical trials on traditional Chinese medicine for heart failure. Chin J Tradit Chin Med. 2020, 35(11): 5694-5697. |
| 93 | Zhang G, Yang Y, Zhao R. Analysis of the characteristics of pediatric drug clinical trials registered in the Chinese Clinical Trial Registry. Chin Pharm. 2020, 31(17): 2055-2060. |
| 94 | Zhang X, Huang L, Tao D, et al. Analysis of the characteristics of radiotherapy clinical trials registered in the Chinese Clinical Trial Registry. J Shanghai Jiao Tong Univ (Med Sci). 2019, 39(07): 789-794. |
