# Supplementary figures and images for: Re-analysis of the current status of clinical trial registration in China
Source: Front Med (Lausanne). 2025 Jan 31;11:1394803. doi: 10.3389/fmed.2024.1394803 (PMC11826807; doi:10.3389/fmed.2024.1394803)

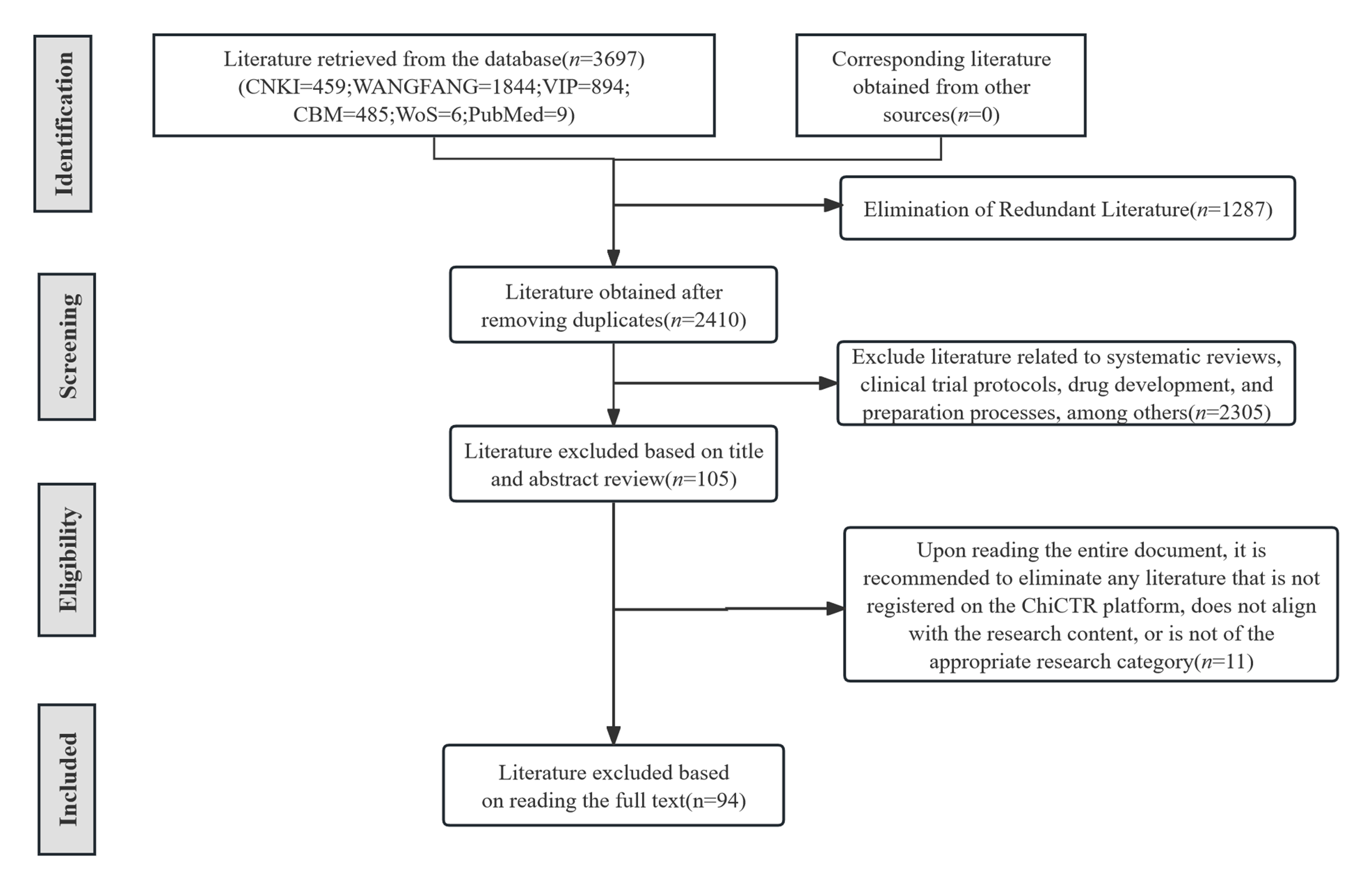

Supplement: Supplementary file 2 [file Image_1.jpeg]
